# HIV/AIDS, Food Supplementation and Livelihood Programs in Uganda: A Way Forward?

**DOI:** 10.1371/journal.pone.0026117

**Published:** 2011-10-12

**Authors:** Jessica E. Yager, Suneetha Kadiyala, Sheri D. Weiser

**Affiliations:** 1 Department of Medicine, Division of Infectious Diseases, University of Washington, Seattle, Washington, United States of America; 2 Vaccine and Infectious Disease Division, Fred Hutchinson Cancer Research Center, Seattle, Washington, United States of America; 3 Poverty, Health, and Nutrition Division, International Food Policy Research Institute, New Delhi, India; 4 Division of HIV/AIDS, San Francisco General Hospital, San Francisco, California, United States of America; 5 Center for AIDS Prevention Studies, University of California San Francisco, San Francisco, California, United States of America; University of New South Wales, Australia

## Abstract

**Background:**

Over the last decade, health, nutrition and policy experts have become increasingly aware of the many ways in which food insecurity and HIV infection negatively impact and reinforce one another. In response, many organizations providing HIV care began supplying food aid to clients in need. Food supplementation, however, was quickly recognized as an unsustainable and incomplete intervention. Many HIV care organizations therefore developed integrated HIV and livelihood programs (IHLPs) to target the root causes of food insecurity.

**Methods and Findings:**

We conducted a qualitative study using in-depth interviews with 21 key informants who worked at seven organizations providing HIV care, food aid, or IHLPs in Kampala, Uganda in 2007-2008 to better understand the impact of IHLPs on the well-being of people living with HIV and AIDS (PLWHAs) and the challenges in transitioning clients from food aid to IHLPs. There was strong consensus among those interviewed that IHLPs are an important intervention in addressing food insecurity and its adverse health consequences among PLWHAs. Key informants identified three main challenges in transitioning PLWHAs from food supplementation programs to IHLPs: (1) lack of resources (2) timing of the transition and (3) logistical considerations including geography and weather. Factors seen as contributing to the success of programs included: (1) close involvement of community leaders (2) close ties with local and national government (3) diversification of IHLP activities and (4) close integration with food supplementation programs, all linked through a central program of HIV care.

**Conclusion:**

Health, policy and development experts should continue to strengthen IHLPs for participants in need. Further research is needed to determine when and how participants should be transitioned from food supplementation to IHLPs, and to determine how to better correlate measures of food insecurity with objective clinical outcomes so as to better evaluate program results.

## Introduction

Policy makers and program implementers concerned with human rights in Africa are increasingly recognizing the complicated relationships between HIV/AIDS and food insecurity. A growing body of evidence indicates that food insecurity, defined as inadequate access to food of sufficient quantity and quality or the inability to acquire food in socially acceptable ways [1], heightens the risk of new HIV acquisition and may lead to worse clinical outcomes in people living with HIV/AIDS (PLWHAs). Specifically, food insecurity leads to increased sexual risk-taking behavior among women in multiple sub-Saharan African nations [2], [3] and acts as a significant barrier to antiretroviral adherence [4]–[10]. Further, recent data from the US indicates that food insecurity has been associated with incomplete HIV RNA suppression [11], worse immunologic status [8], [12], and increased risk of mortality among PLWHA [13].

Conversely, HIV/AIDS is widely recognized as a major determinant of food insecurity. Illness diminishes the ability to engage in livelihood- or food-generating activities, and leaves people vulnerable to food insecurity [14]–[16]. Furthermore, HIV infection often forces a choice between accessing care and treatment or food [9], [17].

Recognition of the interdependence of the HIV/AIDS and food insecurity epidemics has encouraged a number of governmental and non-governmental organizations (NGOs) to integrate food supplementation into their HIV treatment programs [1], [18]–[22]. In April 2001, the United Nations Subcommittee on Nutrition convened a symposium on nutrition, food security and HIV/AIDS where leading international health experts first articulated the critical role of food and nutrition in HIV/AIDS treatment and care. By 2006, UNAIDS formalized this mandate by resolving “to integrate food and nutritional support… as part of a comprehensive response to HIV/AIDS” [22].

Following this lead, food aid programs have started targeting people infected and affected by HIV with short-term interventions providing supplemental food rations lasting between 6 and 12 months. Recent research has found the benefits of food supplementation to include increased physical strength and increased ability to tolerate both antiretroviral medication regimens and activities of daily living [6], [10], [16], [23]. However, while food supplementation can help temporarily alleviate the nutritional deficit that often accompanies food insecurity and advanced AIDS, it may fail to affect other important aspects of food insecurity, including persistent anxiety about the stability of food supplies and the need to obtain food in a socially acceptable manner. As a result, food aid may not address all of the downstream health consequences of food insecurity including ARV non-adherence, ARV treatment interruptions, mental health sequelae, and increased transmission risk behavior. Organizations are therefore beginning to seek more sustainable long-term livelihood-based solutions to address the underlying causes of food insecurity.

In response to this urgent need, programs integrating HIV care and livelihood activities (IHLPs) have developed ad hoc and have far outpaced research evaluating their effects. IHLPs typically support clients in one of three areas: small-enterprise, crop production, or animal husbandry [24]. Small enterprise programs supply small grants to recipients for the development of a business, such as the production and sale of beads, clothing, or other handicrafts. Agriculture and animal husbandry IHLPs generally provide clients with resources and training. Agriculture IHLPs often supply seeds and farm equipment (either individual or shared) as well as ongoing trainings targeting all aspects of crop production, harvesting, and marketing. Animal husbandry IHLPs often provide extensive trainings both before and after delivering an animal and materials to construct an appropriate pen. Ongoing support and training can last for as long as three years in these programs, and may include assistance with sick animals, harvesting equipment or other technical difficulties. IHLPs often also include ancillary services and trainings, such as trainings in hygiene, nutrition and financial management.

Uganda has high rates of both HIV and food insecurity: AIDS is currently the leading cause of death in those between the ages of 15 and 49 [9], and 62% of PLWHAs recently interviewed in urban Uganda stated that household members sometimes or often missed meals [15]. In an attempt to address the synergistic morbidity caused by food insecurity and HIV/AIDS, some innovative grassroots NGOs have initiated IHLPs; yet no published data are available to improve the effectiveness of these programs.

We therefore conducted a qualitative study interviewing key informants who worked at programs targeting integrated HIV care, food supplementation, and livelihood programs in Kampala, Uganda. Key informant interviews are useful in understanding the rationale for programmatic choices in policy implementation and in identifying barriers to and facilitators of specific programmatic efforts. Staff involved in the implementation of IHLPs in Uganda have invaluable knowledge on the successes of and obstacles to improving IHLPs. Using key informant interviews, we aimed to better understand the benefits of IHLPs, challenges in transitioning clients from short-term food assistance to sustainable IHLPs, and the steps needed to ensure that the current focus on the interactions between HIV and food insecurity will lead to effective interventions.

## Methods

### Study Design

We conducted a qualitative study in Kampala, Uganda using in-depth, open-ended key informant interviews with staff at The AIDS Support Organization (TASO) and its collaborators. Planning occurred between September 2007 and February 2008, with data collection in February 2008.

### Study Setting and Participants

TASO is a Ugandan NGO that has directly supported more than 200,000 PLWHAs since it began in 1987. At the time of this study, there were approximately 80,000 TASO clients in Uganda, 20,000 of whom were on antiretroviral therapy (ART). After recognizing the devastating impacts of food insecurity on HIV care, TASO began partnering with organizations that could provide food supplementation to its food insecure clients in 2001. At the time of this study, two of TASO's partner organizations were targeting a total of 16,800 TASO clients to receive food supplementation. The eligibility for food supplementation was determined through evaluation of household composition, employment status and income, possession of different valuable assets, experience of food insecurity and other relevant demographic data. Food was supplied at a household level, and all TASO clients were assumed to be members of a 6 person family; the total number of individuals targeted for food supplementation was therefore 100,800.

Participation in this study was restricted to staff members at TASO or a partner organization who were involved in the development, implementation or oversight of programs related to both direct food aid and livelihood activities. These criteria were chosen for several reasons. First, in a field where few data exist to guide new program development, staff involved in the implementation of these programs have a collective body of experience that is invaluable in assessing the successes of and obstacles to program development. Understanding on-the-ground experiences with these interventions can provide critical data on how best to improve, strengthen, and scale up IHLPs. Second, key informant data provides a system-wide lens through which to evaluate programmatic challenges and successes. By so doing, these data complement and supplement emerging data from program participants. Finally, those interviewed have an important voice in the future development of these programs; understanding their perception of the impact of programs and the future of program development will be important as policy-makers and funders determine a way forward.

Participants were identified through a process of theoretical non-probability sampling [25] at TASO headquarters and at TASO's two primary food aid partner organizations: ACDI/VOCA, which is responsible for implementing USAID's Title II food aid program, and the United Nation's World Food Programme (WFP). We sought key informants who were involved in all aspects of food and livelihood programs. This range included program officers who worked directly with clients, as well as training, monitoring and evaluation, and data specialists. In February 2008, an independent consultant working with ACDI/VOCA facilitated a stakeholders meeting for representatives of food aid and livelihood programs operating in Uganda to discuss issues surrounding the transition of clients off of food supplementation. Further key informants were identified at this meeting in an attempt to capture a broad range of organizations and staff members currently working in Uganda. **Table 1** shows a tabulation of key informants by gender and organization.

**Table 1:** Key informants by gender and organization.

Organization	Male (n = 13)	Female (n = 8)	Total (n = 21)
TASO	4	3	7
ACDI/VOCA	4	2	6
World Vision	2	0	2
AfriCARE	1	0	1
Heifer International	1	1	2
Catholic Relief Services	0	1	1
World Food Programme	1	1	2

### Interviews

Twenty-one individual in-depth open-ended interviews were conducted in English by a US-based physician with experience in qualitative interviewing techniques. The interviews were audiotape-recorded after verbal permission was obtained from participants. The aim of the interviews was to understand how those involved in implementing food supplementation and livelihood programs perceive the integration of HIV care with programs aimed at improving long-term food insecurity. The interview guide, developed together by all three investigators, included semi-structured questions and open-ended prompts. Using this guide, the interviewer ensured that all the domains were covered, while allowing for unanticipated responses. All key informants were asked about their organization's background and aims, methods for targeting clients, exit or transition strategies, monitoring and evaluation, and program sustainability. Given the differences in the missions among the different food supplementation and livelihood programs and the different roles of various key informants within each program, interviews focused on those programmatic issues with which each respondent was most familiar. Interviews lasted between 45 minutes and 3 hours.

### Data Analysis

Interviews were digitally recorded and were transcribed verbatim from audiotapes. Once all interviews were completed, data was reviewed prior to coding to identify emergent themes. Researchers used an integrated deductive and inductive approach [26]: starting with a preliminary code list that resulted from the initial data review, all three researchers compared and categorized the data and further developed appropriate codes based on relevant themes and sub-themes. Using these developed themes, one primary coder manually coded all the data and 25% of interviews were independently coded by a second coder to ensure consistency. An inductive and iterative approach allowed these coders to refine and expand the codes as required by the data. All authors then reviewed the final coding, and coding differences were resolved through discussion and debate. Selected quotes were chosen from interviews to illustrate key themes.

### Ethics Statement

This study was approved by the Institutional Review Boards (IRBs) of TASO, the Uganda National Council on Science and Technology and IFPRI, and interviews were audiotape-recorded after verbal permission was obtained from participants. The individuals interviewed for this study were key informants who were questioned about program processes and not about topics related to their personal health or experiences. The IRBs determined that study procedures posed very minimal if any risk to the participants and that written informed consent was not necessary. Consequently, the IRBs granted a waiver of written informed consent and a waiver of documentation of consent.

## Results

### I. Perceived Benefits of IHLP

Key informants almost uniformly noted that food aid's utility as an intervention was significantly limited by its lack of sustainability. As described by one informant from ACDI/VOCA, “food is not for a lifetime.” In contrast, the majority of informants felt that IHLP interventions were sustainable. Although the key informants we interviewed were not program participants, over half of them noted that, in addition to providing sustainability, IHLPs could potentially improve clients' self-esteem, improve their standing within the community, and reduce the stigma of HIV infection. One TASO staff member described witnessing clients transition from dependence to self-sustenance through these programs:

There is one client who has amazed me, a widow. She has been able…to grow a few things here, to raise goats…As her health improved, her activities…also improved and she is able to look after herself now, and the children who had dropped out of school are back in school. So when you see such an example,…a widow being able to pick up life because she has been given treatment and supported, you say I think this is the way we would want our clients to go and we would really advocate more of these [IHLPs].

Multiple key informants emphasized the critical role of IHLPs in re-establishing an individual's sense of empowerment and self-worth. In describing a prior livelihood program, an ACDI/VOCA informant commented:

When men were asked, ‘What do you think…is the most important thing that you got from this program?’ most of them actually said, ‘I'm able to feed my household all year round and I never used to.’ They would say, ‘Now I can have food for my family. I feel I'm man enough.’

The program staff we interviewed felt that this resulting confidence and competence also affected how individuals were viewed within their communities. According to the country director for one TASO partner:

Some participants...have gained [a] reputation within their communities… Because of the cow, because of the income they are getting, they have been taking on positions of responsibility in their communities, in churches, in schools, on boards… [Now] when there are responsibilities to do, they will say, ‘You know, this one should be the one to conduct this.’

The same key informant explained how this improved standing within the community translated into reduced HIV stigma: “they are now getting less stigmatized in a sense… [the community] can see them managing their own issues, managing themselves, surviving…”

The perception among respondents that IHLPs could help address the underlying causes of food insecurity and could therefore alter the course of illness for PLWHAs led many key informants to argue that all clients coming off food aid should be transitioned to IHLPs. In spite of this belief, however, there was little expertise on how to successfully achieve this transition.

### II. Challenges in the Integration of Food Aid and IHLPs

Perceived barriers to successful transition from food supplementation to IHLPs included a shortage of resources, a lack of criteria for deciding whom to transition and when, and a lack of clarity about both the timing and inputs necessary to render livelihood activities successful.

Approximately half of the key informants interviewed noted that the resources required to transition people from food aid to IHLPs were scarce. Though IHLPs have the potential to render participants self-sufficient and food secure, extensive resources were required to initiate a new participant on an IHLP. These costs could include new livestock, agricultural equipment, or a small grant. At the time of this study, ACDI/VOCA was scaling up the number of direct food aid recipients to 42,000, but they did not have the financial capacity to place all recipients in IHLPs: “we just don't have the resources to get…all the 42,000 people in the agricultural program” (program director, ACDI/VOCA). The program director at World Vision noted the same difficulty: “[on] the issue of giving livelihood support to all the people who are phased off…Funds really are not enough to cater to everyone on the food program.”

Given these funding constraints, key informants struggled to determine which clients were ready to transition from food supplementation into livelihood projects. Many key informants, particularly program directors and those directly involved in monitoring and evaluation, noted the challenge posed by data systems that segregated data on food insecurity status from those data on medical status. TASO, ACDI/VOCA and WFP all assessed clients receiving food supplementation using validated food insecurity measures such as the family's sources of food and valuable resources owned. Additionally, however, TASO clinicians separately evaluated clients' height, weight, body-mass index (BMI) and CD4 count. These clinical data were often recorded, stored and tracked separately from data on food insecurity measures. This segregation made it difficult for program staff to perform a comprehensive interpretation of a client's health status, and key informants specifically involved in monitoring and evaluation activities almost all acknowledged the difficulty in knowing which measures could be used to determine “transition readiness” among PLWHAs. The director of ACDI/VOCA explained:

[We need to decide] whether we need to look at household level indicators or whether we need to look at individual indicators such as clinical status etcetera, or if they should be a combination of both. When we look at the literature, we do not find a lot of examples that can help us handle these issues.

In the absence of a tool that could uniformly determine when clients were ready to transition from food supplementation programs to IHLPs, most organizations provided between 9 and 12 months of food supplementation for their clients, as stipulated by the funding agency. At TASO and World Vision, participants whom clinicians determined had an ongoing medical need for food assistance would sometimes receive ongoing support beyond the standard time period. A senior TASO officer explained,

We give a maximum of 12 months [of food supplementation to] give space to those people who are badly off to get onto the program… If they realize they are getting problems–losing weight, they are having problems with taking their drugs–…if the clinician or the doctor has assessed this person and realized that some of the problems can be solved by getting supplementary feeding then we recommend that person to get back on the food… It is on medical ground; the clinician recommendation is enough.

Organization officials were encouraged by clients who had transitioned themselves off food supplementation as their strength improved. One official noted that “some people are coming up...offering to phase themselves out” (program director, World Vision). A TASO employee similarly described how clients had told her, “I no longer fall sick like I used to… I can now go to the garden and dig; I think its time to really move on.”

Beyond the difficult decisions at the individual level, program officials also struggled with external factors that impacted transition timing. Livelihood activities often required time to produce a sustained benefit, and approximately a quarter of those interviewed cited this lag as a confounding factor in transitioning clients to IHLPs. The amount of time required to develop a successful agricultural enterprise was further extended by seasonal and meteorological variations. A client ready to transition from food aid to agricultural production might need to wait for the seasons to change: “You can't distribute seeds in the dry season because they will not grow” (program director, World Vision).

Program staff at IHLPs also struggled to adapt activities to different locations and situations. The lack of available farming land meant that people in cities and in the north (where ongoing militia activity had displaced much of the population) were unable to rely on agriculture as a means of support. Such constraints often determined which TASO clients were targeted for agricultural activities and which for small-enterprise loans. Explained one TASO official: “What is appropriate for a slum in Kampala is not necessarily what someone in a rural center like Tororo [or] Mbale would benefit from.”

### III. Suggestions for Moving Forward

The informants we interviewed had valuable suggestions about how programs could improve as they move forward. These ideas included strengthening the roles of the community-based organizations and volunteers, strengthening the role of the local government, diversifying activities, and continuing to encourage the transition from food supplementation to IHLP.

TASO staff had used community volunteers to monitor client antiretroviral adherence and provide counseling, and key informants working at TASO were eager to use this workforce to help clients participate in sustainable livelihood activities. Some argued that the best strategy was to mobilize both community volunteers and other PLWHAs as role models. A TASO employee explained: “when we are looking for success stories, these are the clients that we look at so that other people, other clients, can…learn from them.”

Various informants also felt that tighter ties with the government, both central and local, could bolster food supplementation and livelihood programs for PLWHAs. Interestingly, this view was repeated by employees at the World Food Programme, whose institutional focus gave them a different perspective than those working at grassroots organizations with a less robust history of governmental collaboration. Because the UN WFP is mandated to focus on areas considered unstable or insecure, the more politically stable regions of Uganda were recently determined to be ineligible for food aid by the WFP. Regarding the regions from which the WFP was planning to withdraw support, one WFP officer explained, “We are trying to work out with the government to see if any other funding can be solicited so that these people can also be assisted.”

A few informants also felt that diversification of livelihood activities could improve programs' success. A TASO official gave two reasons for this diversification. First, when one activity was not working well, clients would have alternatives that might yield food and income. An informant at ACDI/VOCA elaborated upon this theme:

Because they will grow crops for seven months, you have to…[wait] for two months, seven, eight to nine months before we realize any income. But if they have supplementary enterprises in between here, some other thing to generate income, they can be able to wait and make more money.

Second, diverse activities might produce mutually reinforcing benefits: “We've encouraged for example [people to] have a cow, and the cow dung will help with the garden as manure” (program official, TASO).

Though the majority of informants repeatedly emphasized that integration of food supplementation and livelihood programs was crucial for assuring program and participant success, there was no firm consensus about when clients should be expected to transition fully to livelihood activities. In part, this lack of consensus reflected a dearth of evidence to support any particular timeline. Some informants argued that livelihood activities should begin only after clients had started benefitting from the food and medicine. They believed that that initiating too many different educational components at once would be difficult for participants:

Now after they've taken the food...they have trained on nutrition and hygiene, it is when these other programs can come in. It may not take long for them to start, but initially when you introduce the whole package, it may not work (program coordinator, TASO).

Others suggested that livelihood activities should be started earlier to emphasize the temporary nature of food supplementation. As one TASO official explained, “This time of being able to focus on [IHLP] and really preparing them right from the time they initiate on food is a better approach… Then you know the food is phasing out.”

Finally, informants consistently noted that integrated monitoring and evaluation mechanisms were necessary from the time of program initiation to help determine the program's effectiveness. Without clients' food security and medical status integrated into one data bank, program officials lacked the data to support program changes. Many key informants also struggled with the fact that food insecurity was measured at the household rather than the individual level. This uncertainty regarding appropriate indicators highlighted a key obstacle in developing guidelines for how best to integrate food assistance and IHLPs, and how to measure the effectiveness of both program processes and outcomes. All agreed, however, that such measures were essential to further program development. As one monitoring and evaluations specialist explained, “We need to know why we are collecting the data. We need to clearly define…which data we are collecting and how we are going to collect it.”

## Discussion

In recent years, grassroots organizations, governments, and large multilateral organizations such as the WHO and UN have recognized that addressing food insecurity is a critical component of successful HIV interventions in resource-limited settings [1], [18]-[22]. As a result, there has been increased activity on the ground to jointly address HIV and food insecurity. However, little published data has been available to direct program development.

Food supplementation began as a response to urgent food insecurity among PLWHA. Yet HIV service providers and program staff with experience in the deployment of food aid have come to recognize two significant limitations. First, food supplementation is unsustainable. Second, while food supplementation can ameliorate nutritional deficits, it does not necessarily address other components of food insecurity like persistent anxiety about food access or procuring food in socially unacceptable ways, both of which can lead to worse health outcomes [27]. In light of these limitations, organizations have sought to provide a sustainable alternative to food supplementation by adopting IHLPs. We found consistent agreement among the majority of informants that IHLPs are an important intervention to address long-term food insecurity.

In spite of this agreement, little published data exists to guide future programmatic development. Our key informant interviews underscored several areas urgently in need of further research, summarized in **Table 2**. First, our study is the first that we are aware of to address the difficulties of transitioning PLWHAs from short-term food assistance to long-term IHLPs. A better understanding of this transition could have important implications for programmatic and clinical success. While key informant interviews provide some helpful data to guide future studies and programmatic efforts, in-depth interviews with program clients regarding program experiences and longitudinal studies examining transition timing will be needed as a next step towards understanding these complex issues.

**Table 2:** Critical areas for further research.

Collection of participant data evaluating IHLPs and the transition from food aid to IHLP
Evaluation of IHLPs as a health intervention
Development of better tools for monitoring and evaluation
Cost-effectiveness analysis of food aid and IHLPs
Evaluation of differing impacts of different IHLPs

Questions regarding transition timing highlighted the need for additional research evaluating the role of livelihood programs as health interventions. Again, the experiences of program developers and officers have generated important evidence that IHLPs can have health implications, but there is little data correlating IHLPs with health outcomes. Recently, a pilot study in Kisumu, Kenya found increased household incomes and a trend towards increased CD4 counts in those PLWHAs enrolled in an irrigation intervention [28]. Intervention studies are needed on a larger scale to understand the complex ways in which IHLPs may impact food security, HIV acquisition, HIV treatment outcomes and disease progression.

Third, our research revealed the need for better evaluation tools with which to measure the impact of food supplementation and IHLPs on health outcomes. A recent study evaluating weight gain and HIV disease progression in patients in Uganda found that individuals receiving food supplementation had significant increases in their weight compared with those not receiving food supplementation. However, investigators were unable to detect an impact of food supplementation on HIV progression as determined by WHO stage. The investigators contended that the inability to measure an impact of food aid on WHO disease stage was likely a result of inadequate monitoring and data collection in a “real-life program,” and that a controlled study setting could be expected to yield different results [29]. More thoroughly monitoring changes in food security and HIV/AIDS outcomes as part of food security interventions will be critical to fully understand the range of impacts of food security on health, and to inform intervention development. Furthermore, more studies are needed to understand which markers of HIV disease—clinical stage, viral load, CD4 count—are most useful to follow to accurately determine the impact of interventions targeting food security on HIV outcomes. Longitudinal studies following participants who receive IHLP interventions should capture data related to immune status and function, functional status, nutritional status, food security and morbidity. These data could lead to the development of a prognostic index. Such a tool would help clinicians and program staff allocate limited resources to those PLWHAs for whom food insecurity would be expected to have the greatest impact on health outcomes.

Fourth, data evaluating the cost-effectiveness of food aid and IHLPs are urgently needed to help program officials plan integrated interventions. Our key informants repeatedly noted that cost was a significant barrier in transitioning participants to IHLPs, and those who worked with different livelihood programs emphasized the high costs for some of these programs. However, many IHLPs have tremendous potential to render their participants self-sufficient, and integrate continuity and sustainability into their program models. Heifer International, for example, mandates that all those who receive an animal as part of a livelihood intervention must give at least one of the animal's offspring to a neighbor or other Heifer participant, thereby offsetting future costs to the program. Health metrics models that integrate cost considerations in the evaluation of initial program output versus life years saved could yield important information in determining programmatic priorities for governments and multilateral health and development organizations.

Finally, more studies are needed to evaluate the differing impacts of different livelihood programs. Programmatic opportunities are largely determined by feasibility; our respondents noted, for example, that urban or desert dwellers would not be good candidates for farming programs. However, we are not aware of any research conducted to evaluate the comparative impact of different IHLPs. It is possible, for instance, that agricultural and animal husbandry programs may be better positioned to target food insecurity than other IHLP interventions since they directly impact food production. More research is needed to better understand advantages and disadvantages of different types of IHLP interventions in different settings and populations.

These future research directions could have a significant impact on food policy and program development. Our data corroborated the nascent body of program participant data supporting IHLPs; however, ultimate decisions guiding program focus and development often rest with donors. These donors are generally removed from the ground-level perspective, and further research, as outlined above, will be critical in guiding and justifying program expenditures.

Though many questions remain, our interviews made clear that immediate action is necessary before we have accrued the type of data that could better guide these programs. As research proceeds, governments, multilateral organizations and NGOs should take immediate steps to integrate data systems tracking measurements of health and food insecurity. Furthermore, local and national governments should recognize the important role of IHLPs as a sustainable alternative to food aid.

### Limitations

Our study had several limitations. First, we did not interview program participants, and the key informants we interviewed were all involved in the design and implementation of either food aid programs or IHLPs. Because of their active involvement in these programs, our key informants were invested in these programs and committed to their success. However, as noted above, in light of the lack of available objective data, program officials' perspectives provide a system-wide lens with which to evaluate existing programs, and their views are critical in guiding program efforts. Second, we did not use more diverse qualitative methods to triangulate our results; rather, we aimed to use these key informant data to guide and inform further research [24], including research specifically targeting program participants (unpublished data currently under review). Finally, key informants were all based in Kampala; those program officials in other areas were missed.

### Conclusion

In this qualitative pilot study from Kampala, Uganda, we found extensive agreement among key informants that programs targeting the overlapping epidemics of HIV and food insecurity should better integrate HIV care, food supplementation, and livelihood activities. As governments and organizations come to understand the myriad ways that food insecurity can affect HIV outcomes, both research and programmatic focus must shift to encompass IHLPs as a critical component of HIV care in resource-limited settings.
